# Methionine Augments Antioxidant Activity of Rice Protein during Gastrointestinal Digestion

**DOI:** 10.3390/ijms20040868

**Published:** 2019-02-17

**Authors:** Hui Li, Zhengxuan Wang, Mingcai Liang, Liang Cai, Lin Yang

**Affiliations:** Department of Food Science and Engineering, School of Chemistry and Chemical Engineering, Harbin Institute of Technology, Harbin 150001, China; huili_1024@163.com (H.L.); 18646199358@163.com (Z.W.); hitmcl@163.com (M.L.); feilan1984@sina.com (L.C.)

**Keywords:** methionine, rice protein, antioxidant activity, in vitro digestion

## Abstract

To elucidate the influence of methionine, which is an essential sulfur-containing amino acid, on the antioxidant activity of rice protein (RP), methionine was added to RP (RM). The addition of methionine to RM0.5, RM1.0, RM1.5, RM2.0, and RM2.5 was 0.5-, 1.0-, 1.5-, 2.0-, and 2.5-fold of methionine of RP, respectively. Using the in vitro digestive system, the antioxidant capacities of scavenging free radicals (superoxide; nitric oxide; 2,2′-azinobis (3-ethylbenzothiazoline-6-sulfonic acid) diammonium salt, ABTS), chelating metal (iron), and reducing power were investigated in the hydrolysates of RP and RMs. Upon pepsin-pancreatin digestion, the weakest antioxidant capacity was produced by RP. With the addition of methionine, RMs exhibited more excellent responses to free radical scavenging activities and reducing power than RP, whereas RMs did not produce the marked enhancements in iron chelating activity as compared to RP. The present study demonstrated that RMs differently exerted the free radical scavenging activities that emerged in the protein hydrolysates, in which the strongest scavenging capacities for ABTS, superoxide, and nitric oxide were RM1.5, RM2.0, and RM2.5, respectively. Results suggested that the availability of methionine is a critical factor to augment antioxidant ability of RP in the in vitro gastrointestinal tract.

## 1. Introduction

Reactive oxygen species (ROS) are one of the major risk factors that cause oxidative stress, which can induce a variety of diseases [1]. In this regard, much of what we know about the mechanism of protection against oxidative stress is to scavenge free radicals, which is a useful target for health promotion [2,3,4]. Increasing evidences reveal that oxidative stress can be prevented by dietary antioxidants, highlighting the role of the dietary component in scavenging free radicals to decrease the risk of oxidative damage [5,6,7].

Rice protein is a major plant protein and widely consumed in the world [8]. It has been demonstrated that rice protein has numerous physiological functions, e.g., hypocholesterolemia [9,10,11]. Particularly, recent studies indicate that rice protein can regulate glutathione metabolism via the activation of nuclear factor erythroid 2 (NF-E2)-related factor 2 (Nrf2), a component of endogenous antioxidant defense, thereby resulting in an antioxidative response to attenuate ROS-induced oxidative damage [12,13,14]. Thus, the association of rice protein consumption with a reduced risk of oxidative stress is evident.

The biological utilization of a protein is primarily dependent on its amino acid profile and gastrointestinal digestion [15,16,17]. In light of this view, a convincing explanation that the cholesterol-lowering effect of rice protein lies in its amino acid profile has been provided by Yang and Kadowaki, in which the supplementation with methionine did not abolish the hypocholesterolemic effect of rice protein (RP), suggesting that methionine might play a major role in regulating hypocholesterolemic action [18]. To support, some results showed that the supplementation of methionine did not augment oxidative stress induced by high cholesterol and a methionine deficiency could induce hypercholesterolemia and lipoprotein susceptibility to peroxidation [19,20], highlighting a role of methionine in cholesterol metabolism and antioxidant activity.

Methionine is an essential sulfur-containing amino acid [21,22], which plays a critical role in glutathione synthesis and exerts an antioxidant effect in various physiological reactions [23,24]. Furthermore, some studies have revealed that oxidative damage can be suppressed via the methionine sulfoxide reductase system, suggesting that methionine residues can act as catalytic antioxidants [25]. Thus, the mechanisms by which methionine controls oxidative stress have been attributed to the increase of glutathione synthesis and the stimulation of the methionine sulfoxide reductase antioxidant system [25]. Unfortunately, although a wealth of data regarding the importance of methionine in regulating cholesterol metabolism and stimulating endogenous antioxidant activity exists, the correlation between the methionine availability and the antioxidant activity of rice protein is far from clear. Particularly, up to now, the evidence concerning whether or how the addition of methionine to rice protein can enhance its antioxidant effect and exert free radical scavenging activity in the gastrointestinal tract is lacking.

Thus, the present study was conducted to obtain insight into the influence of methionine availability on the antioxidant capacity of rice protein in an in vitro gastrointestinal system. The key questions addressed are: (1) does the addition of methionine to rice protein affect the antioxidant response during pepsin-pancreatin digestion and (2) how does methionine regulate the antioxidant activity of rice protein in an in vitro digestive system, including free radical scavenging activity, metal chelating activity, and reducing power?

## 2. Results

### 2.1. ABTS Radical Scavenging Activity

The in vitro ABTS radical scavenging activity of the protein hydrolysates were measured in this study.

**Figure 1:**
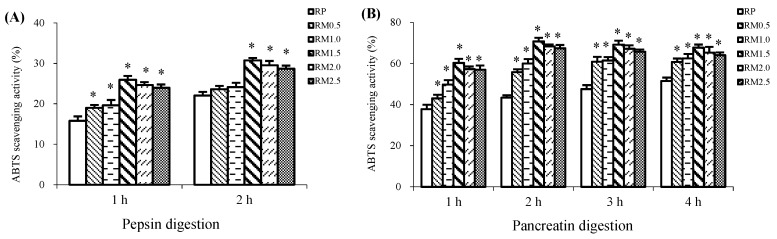
ABTS radical scavenging activities of rice protein (RP) and methionine added to rice protein (RM) hydrolysates during in vitro digestion with pepsin (**A**) and pancreatin (**B**). ABTS, 2,2′-azinobis (3-ethylbenzothiazoline-6-sulfonic acid) diammonium salt. RM, methionine added to rice protein, in which methionine was added to 0.5, 1.0, 1.5, 2.0, and 2.5-fold of rice protein, respectively. RP, rice protein. Values are the means ± SEM (*n* = 3). * *p* < 0.05, in comparison with RP.

During 2 h of pepsin digestion, compared with RP, the addition of methionine increased the ABTS radical scavenging activity. As shown in Figure 1A, the ABTS radical scavenging activity of RM1.5, RM2.0, and RM2.5 was much greater than that of RM1.0, RM0.5, and RP (*p* < 0.05) (RM; methionine added to rice protein). RP had the lowest ABTS radical scavenging activity. The results indicated that the ABTS radical scavenging activity decreased in the order RM1.5 > RM2.0 > RM2.5 > RM1.0 > RM0.5 > RP, and no significant differences were present among RM1.5, RM2.0, and RM2.5 (*p* > 0.05).

After the 2 h pepsin digestion, the ABTS radical scavenging activities were dramatically stimulated by the addition of methionine, as shown in Figure 1B. Compared with RP, all of the RM hydrolysates had a significantly increased ABTS radical scavenging activity during pancreatin digestion (*p* < 0.05). However, the ABTS radical scavenging activity did not increase uniformly with increased methionine supplementation. RM1.5, not RM2.5, exhibited the best clearance of the ABTS radicals of all of the treatments, but RP had the weakest ABTS radical scavenging activity. Taken together, during in vitro digestion with 2 h of pepsin followed by 4 h of pancreatin, the ABTS radical scavenging activity followed the order RM1.5 > RM2.0 > RM2.5 > RM1.0 > RM0.5 > RP, and no significant differences were found in RM1.0, RM1.5, RM2.0, and RM2.5 (*p* > 0.05).

### 2.2. Superoxide Radical Scavenging Activity

The superoxide radical scavenging activity of the protein hydrolysates during in vitro digestion with pepsin and pancreatin was also determined in this study.

**Figure 2:**
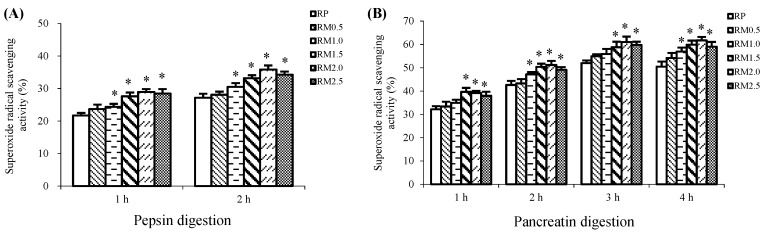
Superoxide radical scavenging activities of rice protein (RP) and methionine added to rice protein (RM) hydrolysates during in vitro digestion with pepsin (**A**) and pancreatin (**B**). Values are the means ± SEM (*n* = 3). * *p* < 0.05, in comparison with RP.

The addition of methionine effectively stimulated the superoxide radical scavenging capacities of the protein hydrolysates, which decreased in the order of RM2.0 > RM2.5 > RM1.5 > RM1.0 > RM0.5 > RP during 2 h of pepsin digestion. As illustrated in Figure 2A, RM2.0 had the strongest superoxide radical scavenging activity among the experimental treatments and RP had the weakest.

The superoxide radical scavenging activity was increased by the addition of methionine during pancreatin digestion as well, showing increases from 7.37% to 22.23% in the RM treatments compared with RP, as shown in Figure 2B. Similar to the findings for the ABTS radical scavenging activity, RM2.5 did not show the strongest superoxide radical scavenging activity, even though the greatest amount of methionine was added. Instead, only RM2.0 showed a significant difference in the superoxide radical scavenging activity after pepsin-pancreatin digestion, whereas RP exhibited the weakest superoxide radical scavenging activity, as shown in Figure 2B. It is noteworthy that during in vitro digestion with pepsin for 2 h and pancreatin for 4 h, RM1.0, RM1.5, RM2.0, and RM2.5 exhibited better clearance of the superoxide radical than RP (*p* < 0.05), but RM0.5 did not show a significant increase over RP (*p* > 0.05).

### 2.3. Nitric Oxide Radical Scavenging Activity

In addition to a strong increase in the superoxide radical scavenging activity, the RM protein hydrolysates also showed an increase in nitric oxide radical scavenging activity.

**Figure 3:**
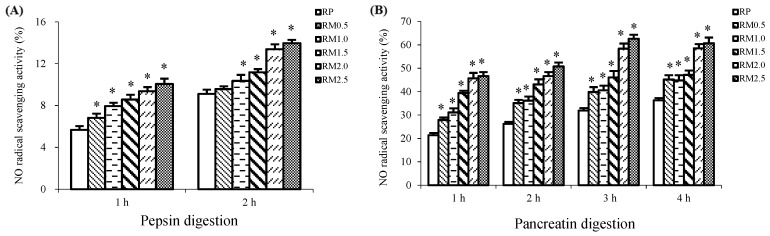
Nitric oxide (NO) radical scavenging activities of rice protein (RP) and methionine added to rice protein (RM) hydrolysates during in vitro digestion with pepsin (**A**) and pancreatin (**B**). Values are the means ± SEM (*n* = 3). * *p* < 0.05, in comparison with RP.

As shown in Figure 3A, the nitric oxide radical scavenging activity of the RM hydrolysate was greater than that of RP during 2 h of pepsin digestion. RM0.5, RM1.0, RM1.5, RM2.0, and RM2.5, respectively, showed 5.05%, 13.72%, 22.61%, 46.87%, and 53.24% increases as compared to RP. The nitric oxide radical scavenging activity decreased in the order RM2.5 > RM2.0 > RM1.5 > RM1.0 > RM0.5 > RP, suggesting that nitric oxide radical scavenging capacity during the 2 h pepsin digestion was dependent on the methionine concentration.

After 2 h of digestion by pepsin, the nitric oxide radical scavenging capacity was also significantly stimulated by the addition of methionine (*p* < 0.05), showing an upward trend in the nitric oxide radical scavenging activity with the increasing level of methionine, as shown in Figure 3B. During in vitro pancreatin digestion, all of the RM hydrolysates exhibited a stronger nitric oxide radical scavenging activity than RP, with significant increases from 23.37% to 67.18% (*p* < 0.05). The best clearance of the nitric oxide radical occurred in RM2.5, and RP had the weakest. As a result, the nitric oxide radical scavenging activity decreased in the order RM2.5 > RM2.0 > RM1.5 > RM1.0 > RM0.5 > RP.

### 2.4. Reducing Power

During pepsin-pancreatin digestion, consistent with the influence on the free radical scavenging activities, RMs, with supplementation of methionine, demonstrated stronger reduction than RP.

**Figure 4:**
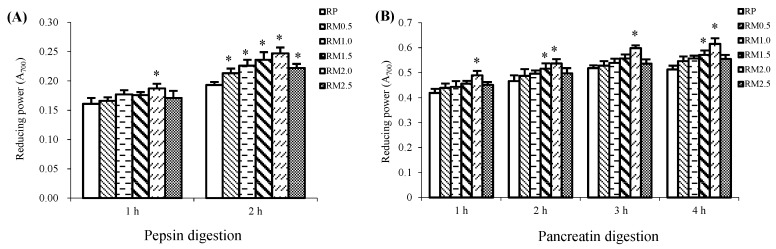
Reducing power of rice protein (RP) and methionine added to rice protein (RM) hydrolysates during in vitro digestion with pepsin (**A**) and pancreatin (**B**). Values are the means ± SEM (*n* = 3). * *p* < 0.05, in comparison with RP.

During the 2 h pepsin digestion, the reducing capacity of RM hydrolysate was greater than that of RP by 10.36% to 27.98%, respectively. Figure 4A shows that RM2.0 had the strongest reducing power and RP the weakest.

Similar to pepsin digestion, pancreatin digestion produced hydrolysates with a greater reducing capacity in the RM treatments than in RP. Figure 4B shows that the reducing activity was enhanced in RM0.5, RM1.0, RM1.5, RM2.0, and RM2.5 with respect to RP by 6.43%, 8.58%, 11.31%, 19.88%, and 8.19%, respectively. During digestion with pepsin for 2 h followed by pancreatin for 4 h, RM2.0 had a significantly higher reducing power than RP (*p* < 0.05).

### 2.5. Fe^2+^ Chelating Activity

In addition to the free radical scavenging activity, the Fe^2+^ chelating activity was also determined in this study.

**Figure 5:**
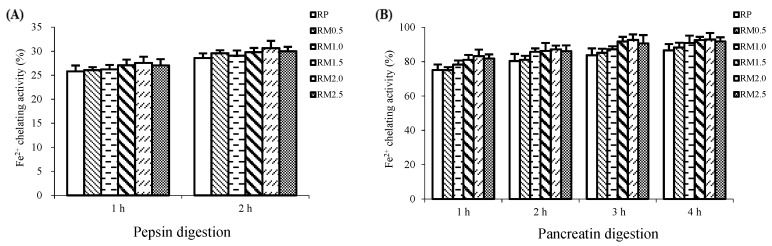
Fe^2+^ chelating activities of rice protein (RP) and methionine added to rice protein (RM) hydrolysates during in vitro digestion with pepsin (**A**) and pancreatin (**B**). Values are the means ± SEM (*n* = 3). * *p* < 0.05, in comparison with RP.

Figure 5A shows that the chelation of Fe^2+^ was slightly stimulated by the addition of methionine by 1.71% to 7.24% compared with RP (*p* > 0.05). During 2 h of pepsin digestion, RM2.0 had the strongest Fe^2+^ chelating activity of the experimental treatments and RP was the weakest. However, pepsin digestion did not produce significant differences in the Fe^2+^ chelating activity in any experimental treatment (*p* > 0.05).

Similar to pepsin digestion, the Fe^2+^ chelating capacity was enhanced to a small extent by pancreatin digestion in the RM treatments compared to RP (*p* > 0.05). Figure 5B shows that all samples had a relatively stable Fe^2+^ chelating activity during pancreatin digestion, with no significant differences among the treatments (*p* > 0.05). During digestion with pepsin for 2 h followed by pancreatin for 4 h, the Fe^2+^ chelating activity was higher in RM0.5 by 1.95%, in RM1.0 by 5.01%, in RM1.5 by 6.95%, in RM2.0 by 7.34%, and in RM2.5 by 6.03% compared to RP. RM2.0 had the strongest Fe^2+^ chelating activity and RP the least, as shown in Figure 5B.

## 3. Discussion

Amino acid composition is a critical factor that affects the antioxidant activity of rice protein [12,13,14]. The availability of sulfur-containing amino acids in the gastrointestinal tract may be more important than other factors with respect to antioxidative status [15,16]. In light of this viewpoint, the effects of methionine on the antioxidant capacity of RP during in vitro pepsin-pancreatin digestion were investigated in this study. To our knowledge, this is the first study that provides convincing evidence that the antioxidant response of RP depends on methionine availability in an in vitro gastrointestinal system.

The process of food digestion is an orchestrated series of bioprocessing operations that involve the breakdown of food, the release of nutrients, their uptake or downstream fermentation before their ultimate removal from the body through defecation [26]. In vitro digestion might be affected by gastric pH, enzymatic activities, gastric emptying rate, etc. In vitro digestion (IVD) modelling is a vivid field of research that shows great promise in facilitating the development of foods and oral formulations based on a better understanding of their digestive fate in the stomach and small intestine as well as downstream ramifications to the gut microbiome [26]. Accordingly, Shani-Levi et al. suggested that IVD models are intended to be more realistic, encompassing various aspects of digestion dynamics (e.g., physiological acid secretion and gastric emptying), mass transport phenomena (i.e., absorption and diffusion), and rheological aspects (i.e., mixing), and including the oral phase, gastric phase, small intestinal phase, and large intestinal phase [26]. As for this study, the key point that we tried to emphasize was the influence of methionine availability on the antioxidant capacity of rice protein in an in vitro gastrointestinal system. Under the present experimental condition, the rice protein used in this study was extracted by alkali treatment and defatted, in which the crud protein (CP) content was 91.6%. Thus, we neglected the possible limitation induced by lipids, mineral, sugar, etc. Instead, we only focused on the process of protein digestion with pepsin-pancreatin, by which amino acids would be released to exert antioxidant activity.

It has been demonstrated that antioxidant activity is primarily exerted by preventing the generation of free radicals, implying that free radical scavenging may be a major contributor to the antioxidant mechanism [27,28]. This suggests that the antioxidant response exerted by RP should first be investigated in terms of the free radical scavenging activity of the hydrolysates in an in vitro digestive system. In this study, the effects of methionine on the free radical scavenging activity of RP were evident. During pepsin-pancreatin digestion, the protein hydrolysates had different free radical scavenging activities.

The ABTS radical is soluble in both aqueous and organic solvents, so the ABTS assay can be used in various media to determine both the hydrophilic and lipophilic antioxidant capacities of dietary components and body fluids [29]. Moreover, the ABTS assay can also be used at different pH levels. Therefore, we chose the ABTS method for this study to investigate the free radical scavenging activity during pepsin-pancreatin digestion in a wide pH range from 2.0 to 8.5 in an in vitro gastrointestinal system.

During pepsin-pancreatin digestion, the hydrolysates with added methionine had better ABTS radical scavenging activities than RP, suggesting that methionine could increase the ABTS radical scavenging activity. This observation is supported by previous findings that methionine reacted directly with peroxides [30]. However, to our surprise, neither RM2.5 nor RM2.0 had the strongest ABTS radical scavenging activity during 2 h pepsin and 4 h pancreatin digestion, but RM1.5, which contained 1.5-fold higher methionine than RP, had the highest ABTS radical scavenging capacity. Interestingly, similar ABTS radical scavenging responses were observed in RM1.0, RM1.5, RM2.0, and RM2.5. At present, the reason for the similar trend in free radical scavenging activity induced by different amounts of methionine is unclear, but the effects of the electron-cloud density on the free radical scavenging activity should be considered. It has been demonstrated that antioxidants can deactivate free radicals by two major mechanisms, hydrogen atom transfer (HAT) and electron transfer (ET) [29]. Similar to an ET-based assay, the ABTS assay detects the ability of a potential antioxidant to transfer one electron to reduce a radical, indicating the reducing capacity of the antioxidants. This viewpoint suggests that the ET mechanism can explain why supplementation with methionine increased the free radical scavenging activity of RP, in which a free radical can accept more electrons to form a more stable free-radical ion. Moreover, the electron-cloud density of the functional group affects its electron-donating activity. A high electron-cloud density increases the electron-donating activity, which in turn increases the scavenging capacity for free radicals [28]. Consequently, this may be the reason that the ABTS scavenging capacity of the RM hydrolysates were greater than that of RP. Additionally, the linkage between the hypersaturated state of the electron-cloud density in RM2.5 and the stability of the ABTS radical scavenging activity could explain the phenomenon that RM2.5 did not show the highest ABTS radical scavenging activity, explaining why the strongest ABTS radical scavenging activity of RM1.5 might be attributed to an optimum level of methionine, which produced the best electron-cloud density in the in vitro gastrointestinal system.

However, because ABTS is a synthetic substrate and represents a “non-physiological” radical source in a biological system, the ABTS assay might be limited in its ability to reflect the free radical scavenging activity of RP. Thus, an improved understanding of the mechanism underlying the cooperation with methionine in a physiological free radical scavenging response is important.

Superoxide is a free radical that is generated in many biological processes. Although the reactivity of superoxide is not high, it is very toxic because it can act as a precursor to other reactive oxygen species, such as hydrogen peroxide and the hydroxyl radical, which oxidatively damages lipids, proteins, and DNA [27,28]. Specifically, superoxide plays an important role in the initiation of lipid peroxidation due to its transformation into the hydroxyl radical and causes various diseases [1]. Accordingly, superoxide scavenging is very important to reduce oxidative stress. Our in vivo studies have demonstrated that the reduction of lipid peroxidation and protein oxidation was possibly consequent upon attenuating the susceptibility to ROS-mediated damage by feeding RP, producing hypocholesterolemic activity [12,13]. The superoxide radical scavenging responses of RP and RM hydrolysates during pepsin-pancreatin digestion in this study support these in vivo findings. The results indicated that the generation of superoxide was effectively suppressed by the RM hydrolysates compared with RP. Significantly, these findings contributed to a favorable explanation to support the view that the addition of methionine could not attenuate the hypocholesterolemic effect of RP [18], which might be involved with the antioxidant response induced by methionine. Obviously, like the ABTS radical scavenging abilities, during 2 h of pepsin and 4 h of pancreatin digestion, RM1.0, RM1.5, RM2.0, and RM2.5 also exhibited a similar superoxide radical scavenging activity. To add to our current findings, in addition to the availability of methionine in the gastrointestinal tract, it is possible that antioxidant activity can also arise from the donation of a hydrogen atom or an electron to superoxide or by a direct reaction with it.

In addition to ROS, reactive nitrogen species (RNS), such as the nitric oxide radical, are also important free radicals in the body [1]. Nitric oxide is an endogenously generated, short-lived free radical that plays a vital role in DNA damage, cytotoxic and inflammatory processes, and causes various diseases [1]. Although there is a wealth of data on the importance of nitric oxide scavenging activity, a correlation between nitric oxide scavenging activity and RP is far from clear. Specifically, relatively little information is available about the nitric oxide radical scavenging activity of methionine in the gastrointestinal tract. After pepsin-pancreatin digestion, a significant finding was made in this study that the nitric oxide radical was effectively scavenged by RP with the addition of methionine. It is noteworthy that the RM hydrolysates showed scavenging activity for the nitric oxide radical dependent on the amount of added methionine and RM2.5, which had the highest methionine concentration, had the strongest nitric oxide radical scavenging activity. These findings were consistent with those of previous studies, suggesting that the sulfur-containing amino acids were particularly sensitive to modification by one or another form of RNS [29]. In addition, the increase of the toxicity of nitric oxide when it reacts with the superoxide radical, forming a highly reactive peroxy nitrate anion, should be taken into account in light of our results. Therefore, RM hydrolysates scavenged RNS more efficiency than RP.

The effect of hydrophobicity on the free radical scavenging activity was also emphasized in this study. Because methionine is among the most hydrophobic amino acids, its hydrophobic nature might be important regarding free radical scavenging during pepsin-pancreatin digestion [16,23]. In support of this view, previous studies have established the critical role of hydrophobicity for the free radical scavenging activity [31,32]. Furthermore, preliminary mechanistic studies have also suggested that the antioxidant activity of a protein is related to its amino acid composition, structure, and hydrophobicity [29,33]. With regard to this, a link between the greater hydrophobicity of RM hydrolysates in this study and a greater free radical scavenging activity is conceivable.

The most frequently suggested mechanisms for the antioxidative effect of dietary proteins are the inhibition of oxidative stress and stimulation of the antioxidative defense status, in which the reducing power has emerged as an important indicator of the antioxidant response [34]. A reducing power assay is often used to evaluate the ability of an antioxidant to donate an electron or hydrogen. In this study, the reducing power assay was used to evaluate the antioxidant ability via the donation of an electron to a free radical and reaction with a free radical, terminating the free radical chain reaction [15,16,17]. Obviously, higher levels of methionine in the RM hydrolysates might confer a greater reducing power than in RP. The increased reducing power of the RM hydrolysates could be attributed to a greater exposure of proton/electron donors, suggesting that RM hydrolysates have a greater capacity to donate an electron than RP. In support of this finding, it has been demonstrated that reducing power is attributed to the chemical characteristics of methionine, cysteine, tyrosine, histidine, lysine, and tryptophan [15,16,31]. Therefore, it is plausible that the addition of methionine promoted the availability of potential electron donors in the in vitro gastrointestinal system, further supporting the finding that RM hydrolysates had a greater free radical scavenging capacity.

It is noteworthy that during in vitro digestion, none of the RM hydrolysates showed a marked enhancement of iron chelating activity compared to RP, despite a slight increase in their chelating activities. It has been recognized that transition metal ions are involved in generation of ROS, resulting in various oxidative damage to lipids and proteins [31,35]. Thus, chelation of iron has been proposed as a potential mechanism for antioxidant activity [15,16,17]. Moreover, some studies reported that the scavenging activity of free radicals was effectively via chelating metal ions, suggesting that metal ion binding affinity might play an important role for protecting against oxidative damage [15,16,17,31,35]. Supportably, in this study, during the in vitro digestion with pepsin and pancreatin, both RP and RM exhibited high iron chelating abilities, indicating a link between iron chelation and efficiencies for scavenging free radicals. However, upon the in vitro pepsin-pancreatin digestion, iron chelation was not significantly affected by the addition of methionine, showing the similar metal chelating activities of RP and RM hydrolysates, from 86.61% to 92.97%. To explain this unexpected phenomenon, the view that acidic (Asp and Glu) and basic amino acids (Lys, Arg, and His) are capable of chelating iron should be taken into account. It has been demonstrated that acidic amino acids and basic amino acids exhibited strong antioxidant ability due to carboxyl and amino groups in the side chains as chelator of metal ions, suggesting that iron chelating activity might be related to the contents of acidic and basic amino acids [31,36]. In light of this view, a satisfactory explanation could be drawn from the present study, suggesting that the similar trend of metal chelating capacities in RP and RM could be attributed to the similar contents of Lys, Arg, His, Asp, and Glu in RP and RM upon pepsin-pancreatin digestion. Therefore, evidence obtained in metal chelating activities further support our view that the antioxidant activity of rice protein is primarily contributed to its digestibility and amino acids available in RP, which in turn determines the acidic and basic amino acids available in the gastrointestinal tract. Hence, the metal chelating activity of RP might be less sensitive to methionine supplementation.

Clearly, the findings of this study suggest that the antioxidant response to RP cannot be merely ascribed to the presence of methionine, which could result in stimulating free radical scavenging and reducing activities. Other amino acids may also come into play to fully explain the antioxidant action of RP. Thus, in addition to methionine, other factors involved in the antioxidant response to RP (e.g., another amino acid profile, special peptide) should be taken into account and remain to be clarified in further studies.

## 4. Materials and Methods 

### 4.1. Protein Sources

Rice protein (RP) extracted from *Oryza sativa* L. cv. *Longjing* 23 (Rice Research Institute of Heilongjiang Academy of Agricultural Sciences, Jiamusi, China) was used as the protein source in the present study. A classical alkaline extraction method (0.2% NaOH) followed by precipitation with an acidic solution was used to prepare RP [8], in which the methionine content was 21.6 mg/g protein. All RP samples were defatted.

Methionine-supplemented RP (RM), in which methionine (Shanghai Aladdin Biochemistry Technology Co., Ltd., Shanghai, China) was added to RP, was used to investigate the effect of methionine on the antioxidant activity of RP. Methionine was added to RP at 0.5-, 1.0-, 1.5-, 2.0-, and 2.5-fold of methionine in RP to produce RM0.5, RM1.0, RM1.5, RM2.0, and RM2.5, respectively. The methionine content was 31.8, 42.4, 53.0, 63.6, and 74.2 mg/g protein in RM0.5, RM1.0, RM1.5, RM2.0, and RM2.5, respectively.

### 4.2. In Vitro Digestion

In vitro digestion of RP, RM0.5, RM1.0, RM1.5, RM2.0, and RM2.5 with pepsin (2 h) and pancreatin (4 h) was performed according to previous studies [8,16]. Briefly, a protein solution (5% *w*/*v*, in distilled water) derived from a defatted protein sample of RP, RM0.5, RM1.0, RM1.5, RM2.0, and RM2.5 was adjusted to pH 2.0 with dilute HCl and incubated at 37 °C with 1% (*w*/*w*, protein basis) porcine pepsin (Sigma, St. Louis, MO, USA). During the pepsin digestion, the digest was sampled at 1 h and 2 h.

After 2 h of pepsin digestion, the digest was adjusted to pH 8.5 with NaHCO_3_ and then treated with 3% (*w*/*w*, protein basis) porcine pancreatin (Sigma, St. Louis, MO, USA). The mixture was incubated at 37 °C for 4 h, and samples were taken at 1, 2, 3, and 4 h of pancreatin digestion.

All samples obtained during the in vitro pepsin-pancreatin digestion were treated with 30% trichloroacetic acid (TCA) and centrifuged at 12,000× *g* for 5 min. After centrifugation, the supernatants (protein hydrolysates) were stored at −20 °C for antioxidant activity analysis.

### 4.3. ABTS Radical Scavenging Activity

The free radical scavenging activity of ABTS was assayed by the method reported in [15,16,17]. Briefly, ABTS was produced by reacting an ABTS (Sigma, St. Louis, MO, USA) stock solution (7 mM) with potassium persulfate (2.45 mM). After a 12–16 h reaction in the dark, the ABTS solution was diluted with 0.2 M phosphate buffer (pH 7.4) to an absorbance of 0.70 ± 0.02 at 734 nm. The protein hydrolysates RP, RM0.5, RM1.0, RM1.5, RM2.0, and RM2.5 were assayed for their ABTS radical scavenging activity. The ABTS radical scavenging activity was determined by mixing 10 µL of sample with 990 µL of the diluted ABTS solution. The absorbance was recorded after a 6 min reaction time. The blank was distilled water instead of sample. The scavenging activity of ABTS was calculated as follows:

Scavenging activity (%) = (A_blank_ − A_sample_)/A_blank_ × 100 (1)

where A_blank_ is the absorbance of the blank and A_sample_ is the absorbance of the sample.

### 4.4. Superoxide Radical Scavenging Activity

The superoxide radical scavenging activity of the protein hydrolysate was determined by the method in [17]. Briefly, 1 mL of sample was mixed with 1 mL of 300 µM nitroblue tetrazolium, 1 mL of 936 µM reduced nicotinamide adenine dinucleotide (NADH), and 1 mL of 120 µM phenazine methosulfate (PMS) in 0.1 M phosphate buffer (pH 7.4). The mixture was incubated at 25 °C for 5 min in the dark, and the absorbance was determined at 560 nm. The blank was distilled water instead of sample. The scavenging activity for superoxide radicals was calculated as follows:

Scavenging activity (%) = (A_blank_ − A_sample_)/A_blank_ × 100 (2)

where A_blank_ is the absorbance of the blank and A_sample_ is the absorbance of the sample.

### 4.5. Nitric Oxide Radical Scavenging Activity

The nitric oxide radical scavenging activity of the protein hydrolysate was measured by a published method [17], with slight modification. Briefly, 950 µL of sample was mixed with 50 µL of 100 mM sodium nitroprusside (SNP) and incubated at 25 °C. After 2.5 h, the mixture was removed and diluted with 1 mL of Griess reagent (1% sulphanilamide and 0.1% *N*-(1-naphthyl)-ethylenediamine dihydrochloride (NED) in 2% H_3_PO_4_) at room temperature for 5 min in the dark. The absorbance was measured at 540 nm. The blank was distilled water instead of sample. The scavenging activity for the nitric oxide radical was calculated as follows:

Scavenging activity (%) = (A_blank_ − A_sample_)/A_blank_ × 100 (3)

where A_blank_ is the absorbance of the blank and A_sample_ is the absorbance of the sample.

### 4.6. Reducing Power

The reducing power of the protein hydrolysate was measured according to a method described by our studies [15,16,17], with slight modification. Briefly, 1 mL of sample was mixed with 1 mL of 0.2 M phosphate buffer (pH 6.6) and 1 mL of 0.1% potassium ferricyanide. The mixture was incubated at 50 °C for 20 min, followed by the addition of 1 mL of 10% TCA. The mixture was centrifuged at 12,000× *g* for 5 min. One milliliter of supernatant was mixed with 1 mL of distilled water and 0.2 mL of 0.1% FeCl_3_. The mixture was incubated for 10 min, and the absorbance was measured at 700 nm. A higher absorbance of the reaction mixture was indicative of a greater reducing power.

### 4.7. Chelating Activity on Fe^2+^

The ability of the protein hydrolysate to chelate Fe^2+^ was investigated using the method in [15,16,17]. A diluted sample (1 mL) was mixed with 0.1 mL of 0.2 mM FeCl_2_ and 0.2 mL of 5 mM ferrozine. The mixture was reacted for 10 min at room temperature and the absorbance was read at 562 nm. The blank was distilled water instead of sample. The chelating activity of Fe^2+^ was calculated as follows:

Chelating activity (%) = (A_blank_ − A_sample_)/A_blank_ × 100 (4)

where A_blank_ is the absorbance of the blank and A_sample_ is the absorbance of the sample.

### 4.8. Statistical Analysis

Data are expressed as the mean ± SEM. Differences between groups were examined for statistical significance using one-way analysis of variance (ANOVA) followed by the least significant difference test. The criterion for significance was *p* < 0.05.

## 5. Conclusions

This study is the first to demonstrate the impact of methionine on the antioxidant activity of RP. Using an in vitro digestive system, this study provided convincing evidence that methionine is a critical factor that augments the free radical scavenging and reducing activities of RP, but the metal chelating capacity of RP is less sensitive to the addition of methionine. The novel findings observed in this study were that RM protein hydrolysates had different free radical scavenging activities, suggesting that the antioxidant response induced by RP depends on methionine availability in the in vitro gastrointestinal system. Thus, the significance of this study is that the methionine availability of rice protein will be a useful target for health promotion through exerting antioxidant activity against ROS-induced oxidative damage. Clearly, the relevance of methionine regarding the antioxidant response and the precise antioxidative defense mechanism of RP requires more detailed investigation in future studies.
